# Meetings of Societies

**Published:** 1910-06

**Authors:** 


### MEETINGS OF SOCIETIES.

### Bristol Medico-Chirurgical Society.

*March 9th*, 1910.

Dr. James Swain, President, in the Chair.

Dr. J. Michell Clarke showed, with the aid of lantern slides, numerous polygraphic tracings illustrating heart affections.—Dr. Carey Coombs discussed the subject.

Mr. C. A. Morton showed **A Stone removed from the Urethra** which had formed round a piece of wood introduced twenty-six years previously, and read notes on the case. (*Vide* p. 127.)

Dr. E. C. Williams read notes on a case of **Leukanæmia** in a boy aged 12, and showed the patient.—The paper was discussed by Drs. Coombs, Clarke, Symes and Waterhouse.

*April 13th*, 1910.

Dr. JAMES SWAIN, President, in the Chair.

The following demonstrations of clinical methods were given :—

Dr. WATSON WILLIAMS demonstrated the use of the **Killian-Brüning's method of direct Laryngoscopy and Œsophogoscopy.**

Mr. C. F. WALTERS demonstrated the use of the **Cystoscope** with bladder models and water-colour drawings.

Mr. T. CARWARDINE showed a new **Speculum** for direct female cystoscopy.

Drs. MICHELL CLARKE, CAREY COOMBS and C. E. K. HERAPATH demonstrated the **Cardiac Polygraph.**

Dr. OGILVY showed a form of **Magnifying Spectacle** which avoids undue convergence.

Mr. C. H. WALKER showed **Worth's Amblyoscope** and **Edridge Green's Lamp for Colour Testing.** (*Vide* p. 175.)

Dr. STACK demonstrated (1) a method of applying **Croft's Splints,** (2) of fixing the elbow joint in an acutely flexed position, and (3) the apparatus for osteoclasia and the method of its use.

---

*May 11th*, 1910.

Dr. J. MICHELL CLARKE in the Chair.

Mr. H. F. MOLE showed a patient in whom a large post-operative **Ventral Hernia was cured by the insertion of a silver wire filigree.** The filigree was placed on the external surface of the peritoneum. There was a gap four inches wide in the muscles, which could only be very partially closed over the filigree by suturing.—Mr. C. A. MORTON mentioned a case in which he had used filigree for the cure of a large hernia extending as low as the knee in a woman. The muscular gap was about two inches across and the muscular and fascial layers extremely thin. The filigree had been placed external to the sutured muscles and fasciæ, and there had been no recurrence.—Mr. MOLE thought the deeper the filigree was placed the better.

Dr. J. O. SYMES showed a man with **Pernicious Anæmia and Ataxic Paraplegia.** The anæmia had greatly diminished under treatment during the last twelve months, but ataxic paraplegia had gradually developed.—Dr. MICHELL CLARKE remarked that in some cases of pernicious anæmia ataxy with excessive reflexes developed, and in others the reflexes were lost. The former group with spastic trouble lasted as long or longer than the latter. Both were to be looked upon as produced by the action of the poison which produced the anæmia acting upon the spinal cord ; but similar cord changes might occur without the anæmia.—Dr.

SYMES agreed that the cases with lost reflexes progressed more rapidly. It was an interesting question whether the patient shown would ultimately develop into a condition with anæsthesia and flaccid paralysis. Such a course of the disease with three stages had been described, though he had not himself met with it. He had seen many cases with loss of reflexes.

Professor WALKER HALL showed, in the absence of Dr. Watson Williams, specimens obtained from a case of **Malignant Growth (Nephroma) of Ear simulating Aural Polypus.** The patient had been under the care of Dr. Williams. The man had a growth in the ear and swellings on the buttock, thorax and parietal bone. The aural growth was partially removed more than once, and in the last portion removed the speaker had found cells which resembled supra-renal cells. Later a *post-mortem* was obtained, and a large tumour found beneath the kidney capsule on one side. This was a mixed nephroma and hypernephroma. The temporal and parietal bones, both affected, were shown, and numerous sections of the tumours.

Dr. EMRYS-ROBERTS gave a demonstration of **Fleming's Modification of Wassermann's Reaction.**—Professor HALL congratulated the speaker upon the lucidity of his demonstration. One could not yet say that the test was quite reliable, but it gave fairly presumptive evidence of syphilis when positive.—Dr. NIXON said that the test might be more useful in discovering whether a patient was cured of syphilis than for the diagnosis of borderland cases, but the difficulties in the former direction were greater than in the latter.—Dr. WILLS asked if the test was reliable in the earliest stages of syphilis.—Dr. CLARKE alluded to the fact that the reaction was obtained in tabes when too late for anti-syphilitic treatment to be of use.

Fleet-Surgeon W. E. HUME, R.N., read a paper on **Public Health Administration in Hamburg.** He gave an interesting account of the thoroughly modern and scientific methods in use in Hamburg for obtaining a pure water supply, for disposing of sewage, for inspecting and slaughtering animals for food, for vaccination, and for the notification of infectious diseases.

Mr. C. CORFIELD read a paper on **Ether or Chloroform as a Routine Anæsthetic.** The routine use of ether, wherever possible, was strongly advocated by reason of its greater safety. The respiratory troubles attributed to ether were avoidable by the use of the open method of administration. The use of ether should be avoided, however, when lung disease was present.—Mr. S. V. STOCK said he had collected records of the deaths under anæsthetics which had occurred to the present date in 1910, fifty-three in number, and found that twenty-eight of the patients were anæsthetised with chloroform, ten with chloroform and ether, three with spinal injections, one with ethyl chloride, one with chloroform and oxygen. This series formed a strong indictment against chloroform. Armstrong had found that in 2,500 ether administrations pulmonary complications had ensued within forty-eight hours in fifty-five cases (rather over 2 per cent.). The speaker advocated the administration of ethyl-chloride, of which 1 c.c. was a sufficient quantity to use, followed by ether. This was better than gas and ether, for it avoided cyanosis and the hypersecretion due to cyanosis. The danger of over-stimulation from ether had to be remembered, and for long and severe operations he thought after using ether for a time then chloroform should be given, and finally, when the operation was being concluded, ether should again be given.—Mr. CORFIELD agreed that ethyl-chloride and ether were better than gas and ether, but he had usually given 3 or 4 c.c. of ethyl-chloride. He would not give ether in cases of phthisis, and had seen bad results from such administrations.

J. LACY FIRTH.
J. A. NIXON, *Hon. Sec.*

---

